# Evaluation of SBA training programme in Karnataka

**DOI:** 10.1186/1753-6561-6-S5-O21

**Published:** 2012-09-28

**Authors:** Devina Bajpayee, Mohan Raju

**Affiliations:** 1 ICRA Management Consulting Services Limited, New Delhi, India; 2 Karnataka Health Systems Resource Centre, Bangalore, India

## Introduction

Skilled birth attendance (SBA) refers to the care provided to a woman and her newborn during pregnancy, childbirth and immediately after birth by an accredited and competent health care provider who has at her/his disposal the necessary equipment and the support of a functioning health system, including transport and referral facilities for emergency obstetric care. The skilled attendant is at the centre of the continuum of care. At the primary health care level, she/he will need to work with other care providers in the community, such as traditional birth attendants and social workers. She/he will also need strong working links with health care providers at the secondary and tertiary levels of the health system. The government of Karnataka is committed to ensure universal coverage of all births with skilled attendance and in this direction has introduced SBA training. This study was mandated with the intent to evaluate the implementation of SBA training for staff nurses and auxiliary nurse-midwives (ANM) across the 30 districts of Karnataka.

## Methods

A multistage, stratified, random sampling with 95% confidence level and 5% confidence interval was used wherein all the primary units (SBA trained personnel) of the sampling frame were divided into two secondary groups namely SBA trained staff nurses and SBA trained ANMs. These secondary groups were further divided into district wise groups. At the district level, SBA trained staff nurses were divided into four categories depending upon the institutional category she was coming from. Similarly SBA trained ANM were divided into five categories based on her institution level. Study sample was taken from these groups based upon the sample size. Overall a sample of 706 SBAs was picked for their knowledge and skill evaluation.

## Results

In the knowledge test, we found that overall 82% of the SBAs (SN and ANM together) had knowledge about various aspects of antenatal, intranatal, postnatal and infection control practices, with SNs having relatively higher knowledge than ANMs (83% vs. 80%). Skill evaluation of SNs and ANMs revealed statistically significant differences in their performances. On the aspect of care of the mother and child, the performance of ANMs was better than that of the SNs; the reason for this could be the postnatal visits and observations by ANMs which are integrated into their job profile. There is an association between knowledge and skills acquired across all categories among both the groups of SBA, but this association was not found to be statistically significant.

## Discussion

In order to reach the stated goal of complete coverage for all childbirths, a state level policy on SBA clearly identifying the role of each level of public healthcare facility and healthcare provider is needed. The services delivered at private institutions should be included in the ambit. Based on the state policy, the role and scope of work for the ANM posted at a subcentre should be clearly defined. SBA training for ANMs may then be customized to lay greater stress on antenatal care, identification of complications during labour and childbirth and their prompt referral to the suitable level of facility.

## Competing interests

None declared

## Funding statement

None declared

